# Observation of the spin-polarized surface state in a noncentrosymmetric superconductor BiPd

**DOI:** 10.1038/ncomms13315

**Published:** 2016-11-07

**Authors:** Madhab Neupane, Nasser Alidoust, M. Mofazzel Hosen, Jian-Xin Zhu, Klauss Dimitri, Su-Yang Xu, Nagendra Dhakal, Raman Sankar, Ilya Belopolski, Daniel S. Sanchez, Tay-Rong Chang, Horng-Tay Jeng, Koji Miyamoto, Taichi Okuda, Hsin Lin, Arun Bansil, Dariusz Kaczorowski, Fangcheng Chou, M. Zahid Hasan, Tomasz Durakiewicz

**Affiliations:** 1 Department of Physics, University of Central Florida, Orlando, Florida 32816, USA; 2 Joseph Henry Laboratory and Department of Physics, Princeton University, Princeton, New Jersey 08544, USA; 3 Theoretical Division and Center for Integrated Nanotechnologies, Los Alamos National Laboratory, Los Alamos, New Mexico 87545, USA; 4 Center for Condensed Matter Sciences, National Taiwan University, Taipei 10617, Taiwan; 5 Department of Physics, National Tsing Hua University, Hsinchu 30013, Taiwan; 6 Institute of Physics, Academia Sinica, Taipei 11529, Taiwan; 7 Hiroshima Synchrotron Radiation Center, Hiroshima University, 2-313 Kagamiyama, Higashi-Hiroshima 739-0046, Japan; 8 Centre for Advanced 2D Materials and Graphene Research Centre, National University of Singapore, Singapore 117546, Singapore; 9 Department of Physics, National University of Singapore, Singapore 117542, Singapore; 10 Department of Physics, Northeastern University, Boston, Massachusetts 02115, USA; 11 Institute of Low Temperature and Structure Research, Polish Academy of Sciences, 50-950 Wroclaw, Poland; 12 Condensed Matter and Magnet Science Group, Los Alamos National Laboratory, Los Alamos, New Mexico 87545, USA; 13 Institute of Physics, Maria Curie - Sklodowska University, 20-031 Lublin, Poland

## Abstract

Recently, noncentrosymmetric superconductor BiPd has attracted considerable research interest due to the possibility of hosting topological superconductivity. Here we report a systematic high-resolution angle-resolved photoemission spectroscopy (ARPES) and spin-resolved ARPES study of the normal state electronic and spin properties of BiPd. Our experimental results show the presence of a surface state at higher-binding energy with the location of Dirac point at around 700 meV below the Fermi level. The detailed photon energy, temperature-dependent and spin-resolved ARPES measurements complemented by our first-principles calculations demonstrate the existence of the spin-polarized surface states at high-binding energy. The absence of such spin-polarized surface states near the Fermi level negates the possibility of a topological superconducting behaviour on the surface. Our direct experimental observation of spin-polarized surface states in BiPd provides critical information that will guide the future search for topological superconductivity in noncentrosymmetric materials.

## Abstract (web summary)

A superconducting material containing a topologically non-trivial electronic band structure presents the possibility of realizing Majorana states as well as exotic excitations with potential in quantum computing. Here, the authors evidence the required ingredients in the noncentrosymmetric superconductor BiPd.

## Graphical abstract

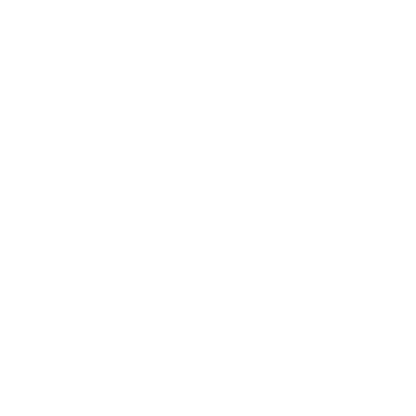

Recently, noncentrosymmetric (NCS) superconductors (SCs) have attracted considerable research interest due to the possibility that they host several exotic states^1^,^2^,^3^. The lack of inversion symmetry creates an asymmetric potential gradient, which may split the electron bands by lifting the spin degeneracy, allowing hybrid-pairing of spin-singlet and spin-triplet states within the same orbital channel^3^,^4^,^5^,^6^. Furthermore, with the advent of topological insulators^7^,^8^,^9^,^10^,^11^,^12^,^13^,^14^,^15^,^16^,^17^,^18^, it was recently proposed that NCS SCs with a strong spin–orbit coupling (SOC) are potential candidates for realizing topological superconductivity^19^,^20^,^21^,^22^, holding promise for hosting protected Majorana surface states^15^,^23^,^24^. But so far, evidence of topological surface states in NCS materials is still lacking. Such convincing evidence may be obtained by angle- and spin-resolved photoemission, which provides an energy- and momentum-resolved probe of the electronic and spin structure.

A NCS system provides a good platform to look for exotic states such as the Weyl semimetal in TaAs^25^ and the topological nodal-line phase in PbTaSe_2_(ref. 26). Another NCS SC BiPd can provide a platform to study the interplay of SOC effects with superconductivity. This compound undergoes a structural transition from *β*-BiPd (orthorhombic) to *α*-BiPd (monoclinic) at 210 °C and then becomes superconducting at the transition temperature (*T*_c_)∼3.7 K (refs 4, 5). In comparison to many other NCS SCs, BiPd is a weakly correlated compound possessing a heavy atom, Bi. Measurements by point-contact spectroscopy^27^ and nuclear quadrupole resonance^28^ indicate a complex gap structure in BiPd, which might be caused by the lack of inversion symmetry^4^,^5^. Recent scanning tunnelling microscopy measurements reveal that the superconducting state of BiPd appears to be topologically trivial, consistent with Bardeen–Cooper–Schrieffer theory with an *s*-wave order parameter^6^. Further momentum and spin-resolved experimental evidence is highly desirable to establish the presence of topological Dirac surface states, and to study the underlying microscopic mechanism of superconductivity. A detailed high-resolution angle-resolved photoemission spectroscopy (ARPES) and spin-resolved ARPES study is necessary to prove or disprove the topological nature of the surface states in the normal state of BiPd. Such a characterization of the normal state is currently missing, but necessary and needed as a first step towards opening a discussion on the relationship between superconductivity and the topological properties of NCS systems.

In this paper, we report the experimental observation of spin-polarized surface states in the NCS material BiPd using ARPES and spin-resolved ARPES. Our experimental results show the presence of a surface state at higher-binding energy, with the Dirac node located at ∼700 meV below the Fermi level. Furthermore, the Dirac node located at ∼650 meV below the Fermi level is also observed for different surface cleaving, providing direct evidence for the noncentrosymmetic crystal structure presented in BiPd. Our measurements suggest that the observed states may be topological in nature. Our results are further supported by our first-principles calculations. The absence of spin-polarized surface states near the Fermi level of BiPd negates the possibility that this system might host topological superconductivity on the surface.

## Results

### Crystal structure and sample characterization

**Figure 1:**
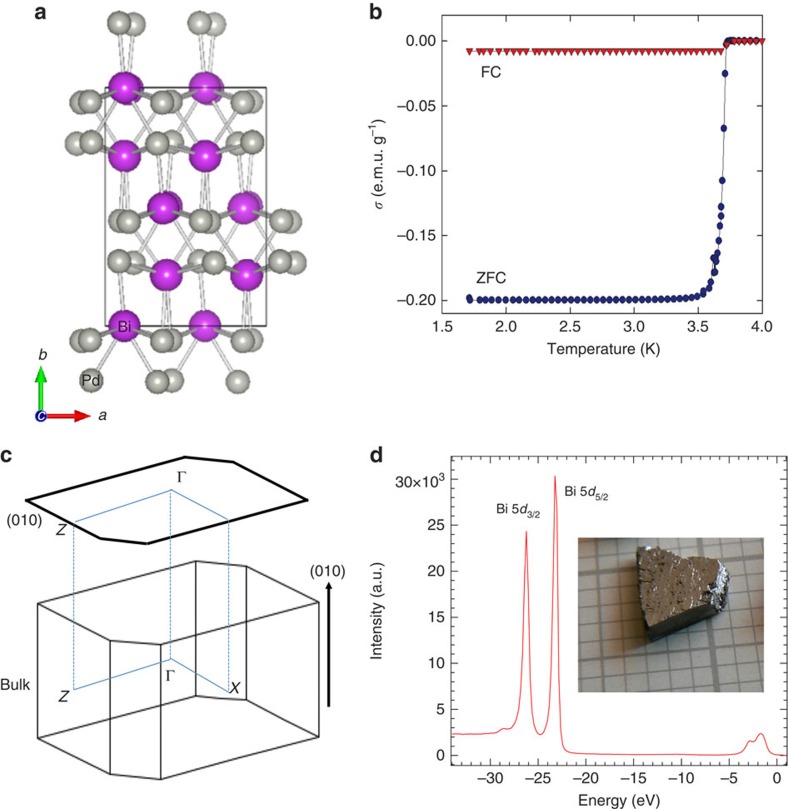
Crystal structure and sample characterization of BiPd. (**a**) Crystal structure of BiPd with the *b* axis shown as its unique axis. It crystallizes in a monoclinic structure at low temperatures. (**b**) The magnetic susceptibility as a function of temperature showing a sharp superconducting transition temperature at ∼3.7 K in the field of 20 Gs. (**c**) Schematic drawing of surface and bulk Brillouin zones, corresponding to a primitive cell, are shown. We note that all electronic structure calculations were performed on a conventional unit cell for the bulk and a supercell for the slab. High-symmetric points are also marked. (**d**) Core level spectroscopic measurement of BiPd showing sharp peaks of Bi 5*d*. The inset shows a photograph of the BiPd sample.

The crystal structure of BiPd at low temperatures (<210 °C) has a monoclinic unit cell with *a*=5.63 Å, *b*=10.66 Å, *c*=5.68 Å, *α*=*γ*=90° and *β*=101° with the *b* axis being as its unique axis (Fig. 1a; ref. 4). Detailed characterization of the single crystals used in our study indicated their high quality (Supplementary Figs 1–7; Supplementary Notes 1–4; Supplementary References). They exhibit a simple metallic behaviour in the normal state and a sharp superconducting transition at *T*_c_∼3.7 K. The low-temperature magnetic susceptibility data is shown in Fig. 1b (see also Supplementary Fig. 1 and Supplementary Note 1 for additional data and discussion). A schematic bulk Brillouin zone is shown in Fig. 1c, where the projected surface along (010) is also illustrated. To experimentally identify its electronic structure, we study the electronic structure of BiPd on the cleaved (010) surface. Figure 1d (a picture of the single crystal measured is displayed in the inset of Fig. 1d) shows momentum-integrated ARPES spectral intensity over a wide-energy window. Sharp ARPES intensity peaks at binding energies *E*_B_∼23 and 26 eV corresponding to the bismuth 5*d*_3/2_ and 5*d*_5/2_ energy levels are observed.

### Electronic structure of BiPd

**Figure 2:**
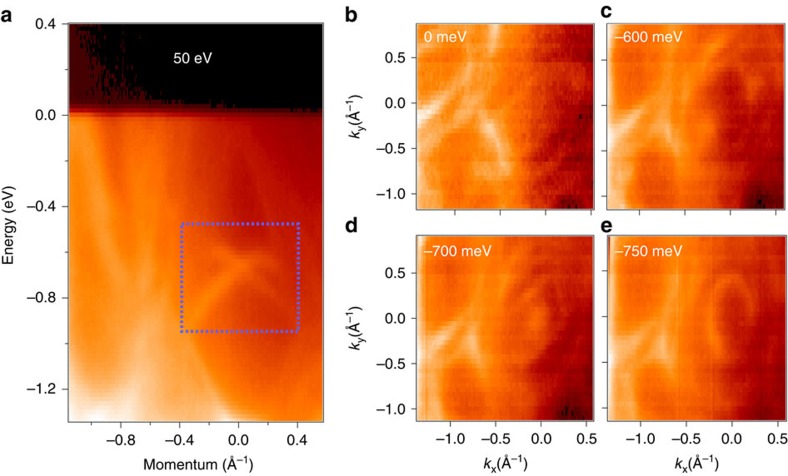
Electronic structure of BiPd. (**a**) Dispersion map of BiPd along the zone centre obtained by using incident photon energy of 50 eV at a temperature of 10 K. The blue rectangle in the binding energy range of ∼500–900 meV shows the linearly dispersive states. (**b**) Fermi surface map. (**c**) Constant energy contour at binding energy of 600 meV shows the intensity map in the region above the Dirac point. (**d**,**e**) The constant energy contours at 700 and 750 meV show the intensity map at around and below the Dirac point, respectively. The values of binding energies are noted on the plots. Data were collected at ALS BL 10.0.1.

We study the overall electronic structure of BiPd using ARPES. Figure 2a shows an ARPES dispersion map in a 1.3 eV binding energy window, where several dispersive bands within the valence band are identified. Moreover, several crossing or metallic bands in the vicinity of the Fermi level are observed. Remarkably, a nearly linearly dispersive Dirac cone-like state is observed at the Brillouin zone centre, showing a Dirac node located at a binding energy of *E*_B_∼700 meV. The Dirac-like state can be observed in the region of the blue rectangle in the Fig. 2a, at the binding energy region of 500−900 meV. At the Fermi level only the metallic bands, but no other Dirac-like linearly dispersive bands are observed. On the other hand, the linearly dispersive Dirac-like bands are found to be in the region of higher-binding energies.

Figure 2b–e shows the Fermi surface and constant energy contour plots, respectively. In the vicinity of the Fermi level, many metallic bands are observed (Fig. 2b). We also study the ARPES measured constant energy contour maps (Fig. 2c–e). At the Fermi level, the constant energy contour consists of many metallic pockets. With increasing binding energy at ∼600 meV, the circular pocket formed by the upper Dirac cone is observed (Fig. 2c). On increasing the binding energy, the size of the pocket decreases and eventually shrinks to a point (the Dirac point) near *E*_B_∼700 meV (Fig. 2d). At even higher-binding energies, the nearly circular pocket formed by the lower Dirac cone is observed (for *E*_B_∼750 meV in Fig. 2e).

**Figure 3:**
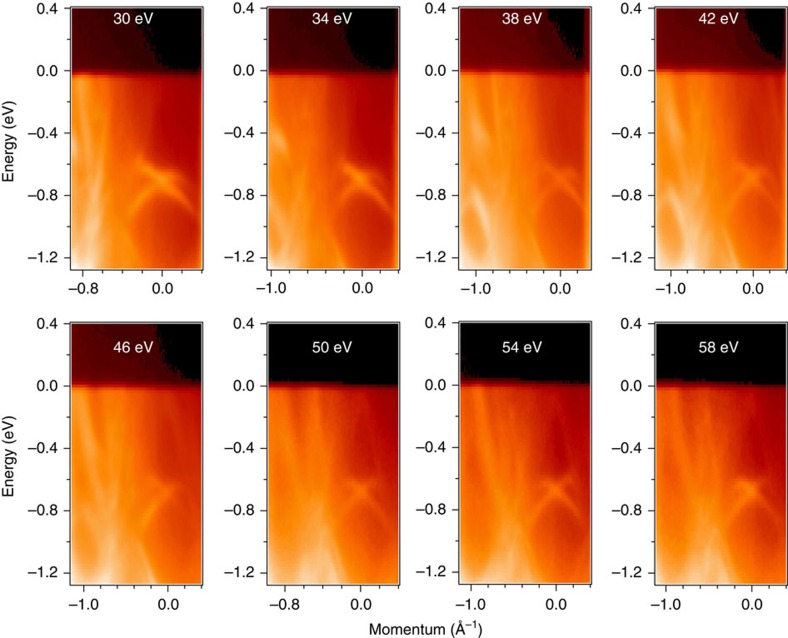
Photon energy-dependent ARPES dispersion maps. The measured photon energies are noted on the plots. The linearly dispersive states at a binding energy of ∼700 meV do not show any dispersion with photon energy, which indicate its two-dimensional nature. These data were collected along 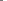
−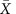 high-symmetry direction at ALS BL 10.0.1 at a temperature of 10 K. Additional photon energy data are presented in Supplementary Information.

To reveal the nature of the states observed in BiPd, we performed photon energy and temperature-dependent ARPES, as well as spin-resolved ARPES measurements that are further complemented by calculations. Figure 3 shows the energy-momentum cuts measured with varying photon energies from 30 to 58 eV with a 4 eV energy step (see Supplementary Figs 2–4 and Supplementary Note 2 for additional ARPES data and discussion). Clear *E*–*k* dispersion of the bulk bands is observed. Remarkably, the dispersion of the linearly dispersive states at high-binding energy (500–900 meV) is found to be unchanged with respect to the varying photon energy, supporting the two-dimensional nature of this state. It is important to note that the Dirac-like two-dimensional states are not found to be perfectly linear in energy-momentum axis. Furthermore, it is important to recall that in real materials such as pure Bi, graphene or topological insulators, the Dirac cones are never perfectly linear over a large energy window yet they can be approximated as linear within a narrow energy window around the Dirac point. This linear part represents the massless dispersion, in contrast to the large effective mass of conventional band electrons in other materials. Moreover, we note that the weak asymmetry of the surface bands with respect to *k* and −*k* is probably coming from the matrix element effects, and from the fact that these measured spectra were slightly off from the 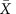
−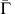
−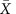 direction.

**Figure 4:**
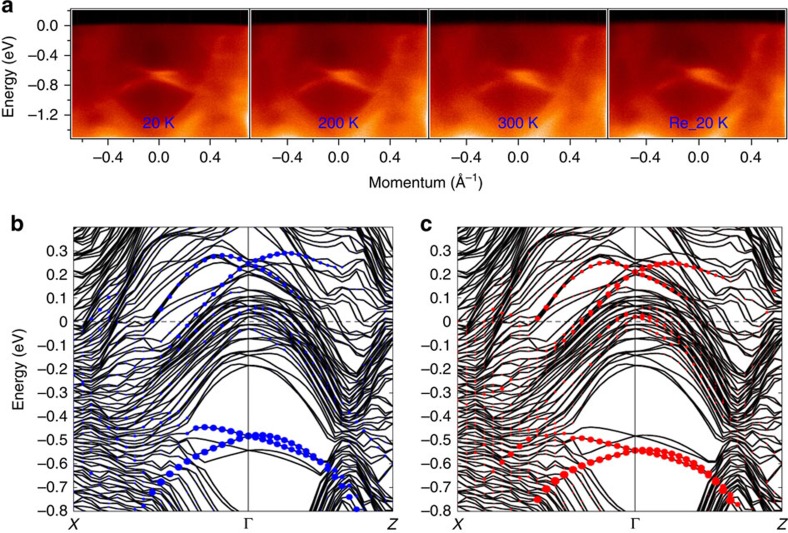
Temperature-dependent spectra and first-principles calculations. (**a**) ARPES energy-momentum dispersion maps measured using a photon energy of 30 eV along the 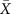
−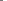
−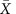 momentum space cut-direction with varying temperatures. The measured values of temperature are noted on the plots. The panel with the note of Re_20K is the spectrum measured after thermal cycling (20→300→20 K). These data were collected at SSRL BL5-4 with a photon energy of 30 eV. (**b**) Slab calculations of BiPd for the top surface and (**c**) the bottom surface along the high-symmetry lines. The blue (red) dots represent surface states for the top (bottom) surface. Details of the calculation method is given in the Method section and additional calculation plots are shown in Supplementary Information.

To test the robustness of the surface state observed in BiPd, we have performed a temperature-dependent measurement as shown in Fig. 4a. On raising the temperature, the Dirac-like surface states survive even at room temperature, which establishes that the Dirac-like surface states are robust to the rise of temperature and potential surface contamination, resulting from increased partial pressure of residual gases released upon heating, see Fig. 4a. Furthermore, the observed Dirac-like states are found to be robust against thermal cycling (20–300–20 K), since lowering the temperature back down to 20 K results in the similar spectra with the strong presence of Dirac-like state features (see the panel of Fig. 4a with the note of Re_20K).

### NCS electronic signature

To better understand the electronic structure observed with ARPES, we perform first-principles calculations of the bulk band structure (Supplementary Fig. 5; Supplementary Note 3) and slab calculations of BiPd using the generalized gradient approximation plus SOC method (see Fig. 4b,c for the calculated electronic structure for the top and bottom surfaces, respectively). We note that the band structure calculations were performed on a conventional unit cell for the bulk system and on a supercell for the slab structure, which leads to folding of the states at the S point to the Γ point. Our slab calculations show that the surface states are predicted to be at the Γ point with the location of the Dirac nodes at ∼0.5 and 0.6 eV below the Fermi level, one of which comes from the top surface and the other from the bottom surface. The Dirac node of the surface state coming from the top and bottom surfaces are located at different binding energies (not degenerate), which comes from the fact that BiPd lacks inversion symmetry.

Since the calculations were made for slab geometry, the obtained band structure always involves bands from two surfaces, one on the top and the other on the bottom of the slab. Experimentally, one can only measure one surface (corresponding to either top or bottom surface of the slab geometry) at a time. In principle, it should be possible to measure either the top or bottom surface on the two sample pieces based on the cleaving. Therefore, depending on whether the cleaved surface is a top or bottom one, the location of the Dirac point energy is predicted to be different. Experimentally, the location of the Dirac point energy is found to be different based on the cleaving either the top or the bottom surface, in agreement with our calculations. Actually, we experimentally observed the different Dirac point energy for top and bottom surface cleaving. For the top surface the Dirac point is ∼700 meV, while for the bottom surface cleaving Dirac point is ∼650 meV from the Fermi level (Supplementary Fig. 4; Supplementary Note 3). We note that such two terminations of the crystal surface in BiPd are directly observed by STM topographic image^6^.

**Figure 5:**
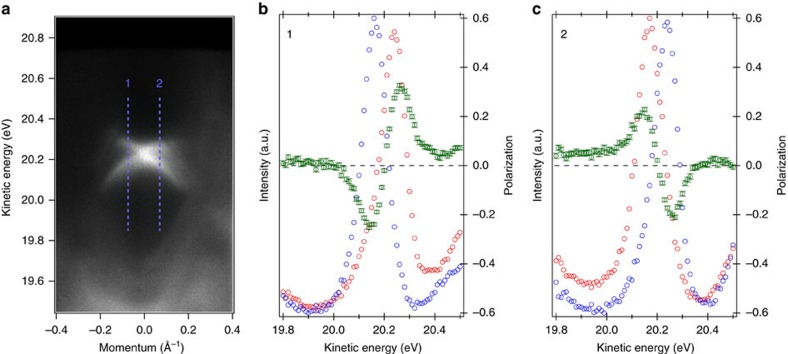
Spin-resolved ARPES results. (**a**) Spin-integrated ARPES spectrum of surface states. The blue dashed lines and number indicate the momentum positions at which SR-ARPES measurements were carried out. (**b**) Spin-resolved energy-dispersive curvess (blue and red circles for up and down spin) and the in-plane spin polarization (red circle with error bar -right axis) at momentum position #1. (**c**) Same as **b** for momentum position #2. Helical spin-texture is observed in BiPd. Error bars represent the experimental uncertainties (s.d.) in determining the spin polarization. Spin-resolved measurements were carried out with photon energy of 25 eV and temperature of 20 K at Beamline-9B of the Hiroshima Synchrotron Radiation Center (HiSOR), Hiroshima, Japan.

To further understand the nature of the observed Dirac-like band, we measured the in-plane spin polarization or spin-texture properties of BiPd. Spin-resolved ARPES measurements were performed on the top-cleaved surface of BiPd. Two spin-resolved energy-dispersive curves are shown at momenta of ∼±0.1 on the opposite sides of the Dirac-like dispersion. The blue dash lines on the ARPES dispersion map shown in Fig. 5 mark the approximate momentum positions for the spin-resolved energy distribution curves. The spin-resolved energy-dispersive curves and corresponding net spin polarization are shown in Fig. 5b,c for momentum positions #1 and #2. The obtained spin data presented in Fig. 5b,c show observable net spin polarization. Importantly, the observed spin polarization at one branch of the cone is opposite with that of another cone, thus confirming the spin-momentum locking behaviour. Spin-resolved ARPES data reveals a characteristic helical spin-texture, suggesting the possible topological origin of the states we observed. However, we caution that two-band crossings at a time-reversal invariant momentum should have an opposite value of the spin polarization independent of any topological invariant.

## Discussion

The experimental realization of the topological insulator phase in a NCS crystal structure is an object of intense research. Such a system may be utilized in testing several proposed exotic phenomena, such as crystalline-surface-dependent topological electronic states, pyroelectricity and natural topological *p*–*n* junctions^29^. Recently, the first-principles calculations predicted III-Bi to be an inversion asymmetric topological insulator with large bandgap possessing intrinsic topologically protected edge states and forming quantum spin Hall systems^29^, but these have not yet been realized experimentally. At the same time, the proposal of topological insulating nature in an inversion asymmetric compound BiTeCl still remains under debate^30^,^31^. Furthermore, the small bulk bandgaps of the realized inversion asymmetric topological insulators severely limit the manipulation and control of the topological surface states.

We note that BiPd consists of many bands near the Dirac-like states, which makes this system very complicated with no global bandgap in the normal state. The topology in BiPd is not well defined such as in Bi_2_Se_3_. In ARPES experiments, the samples were cleaved and measured in an ultrahigh vacuum environment that kept its surface clean during the measurements. The surface cleaving generates surface potential due to the surface charges, which may develop a large effective pressure along the *b* (perpendicular to the surface, see Fig. 1a) direction and thus drive the crystal (possibly the several top layers) into the topological insulator phase. Our calculations on strained BiPd show the bulk bandgap opening, (Supplementary Fig. 7; Supplementary Note 4). This study will enable further discussion on the theoretical origin of the topological order in NCS materials.

BiPd is a SC below *T*_c_∼3.7 K. Since the spin-polarized surface state is located at high-binding energy, it negates the topological superconductivity behaviour on the surface at its native Fermi level. However, by electrical gating or surface deposition, the Fermi level can be tuned near the Dirac surface state, which provides an opportunity to realize the topological superconductivity in this noncentrosymmetic material.

## Methods

### Crystal growth and characterization

Single crystals of BiPd were grown by a modified Bridgman method as described elsewhere^4^. The crystals were characterized by means of X-ray diffraction, energy-dispersive X-ray spectroscopy, magnetic susceptibility, electrical resistivity and heat capacity measurements, using standard commercial equipment.

### Electronic structure measurements

Synchrotron-based ARPES measurements of the electronic structure were performed at the Advanced Light Source (ALS), Berkeley at Beamline 10.0.1 and Stanford Synchrotron Radiation Lightsource (SSRL) at Beamline 5–4 both equipped with a high efficiency R4000 electron analyser. The energy resolution was set to be better than 20 meV for the measurements with the synchrotron beamline. The angular resolution was set to be better than 0.2° for all synchrotron measurements. Samples were cleaved *in situ* and measured at 10–80 K in a vacuum better than 10^−10^ torr. They were found to be very stable and without degradation for the typical measurement period of 20 h.

### Spin-resolved ARPES measurements

Spin-resolved ARPES measurements were performed at the ESPRESSO end station installed at Beamline-9B of the Hiroshima Synchrotron Radiation Center (HiSOR), Hiroshima, Japan, equipped with state-of-the-art very low-energy electron diffraction spin detectors utilizing preoxidized Fe(001)-p(1 × 1)-O targets^32^,^33^. The two spin detectors are placed at an angle of 90 ° and are directly attached to a VG-Scienta R4000 hemispheric analyser, enabling simultaneous spin-resolved ARPES measurements for all three spin components. We also measured the *x* and *z* component of the spin that shows negligible spin polarization.

### First-principles calculations

The first-principles calculations were based on the generalized gradient approximation^34^ using the projector augmented-wave method^35^, as implemented in the VASP package^36^,^37^. The experimental crystallographic structure was used^38^ for the calculations. The SOC was included self-consistently in the electronic structure calculations with a 6 × 4 × 5 Monkhorst-Pack *k*-mesh. To simulate surface effects, we used 1 × 5 × 1 supercell for the (010) surface, with a vacuum thickness larger than 20 Å.

### Data availability

All relevant data are available from the corresponding author upon request.

## Additional information

**How to cite this article:** Neupane, M. *et al*. Observation of the spin-polarized surface state in a noncentrosymmetric superconductor BiPd. *Nat. Commun.* **7,** 13315 doi: 10.1038/ncomms13315 (2016).

**Publisher's note:** Springer Nature remains neutral with regard to jurisdictional claims in published maps and institutional affiliations.

## Supplementary Material

Supplementary Information Supplementary Figures 1-7, Supplementary Notes 1-4 and Supplementary References.
