# Supplementary material for: Observation of the spin-polarized surface state in a noncentrosymmetric superconductor BiPd
Source: Nat Commun. 2016 Nov 7;0:13315. doi: 10.1038/ncomms13315 (PMC5103058; doi:10.1038/ncomms13315)
Supplement: Supplementary Information — Supplementary Figures 1-7, Supplementary Notes 1-4 and Supplementary References. [file ncomms13315-s1.pdf]

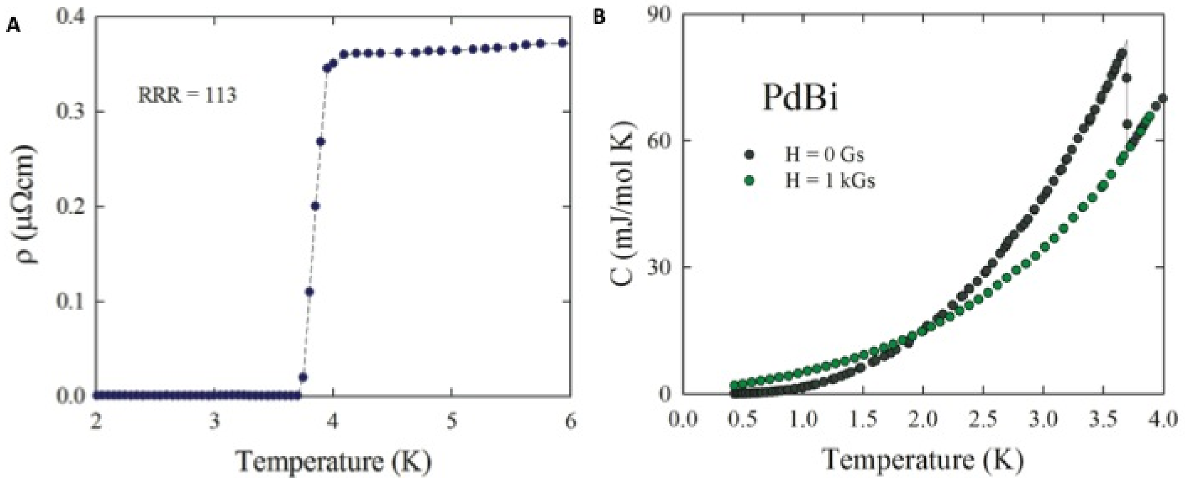

Supplementary Figure 1: Sample characterization of BiPd. (a) Temperature dependence of electrical resistivity within (010) plane showing a sharp transition at $T_c \sim 3.7$ K. (b) The temperature dependence of the specific heat capacity with no field (black filled circles) and with field (red filled circles).

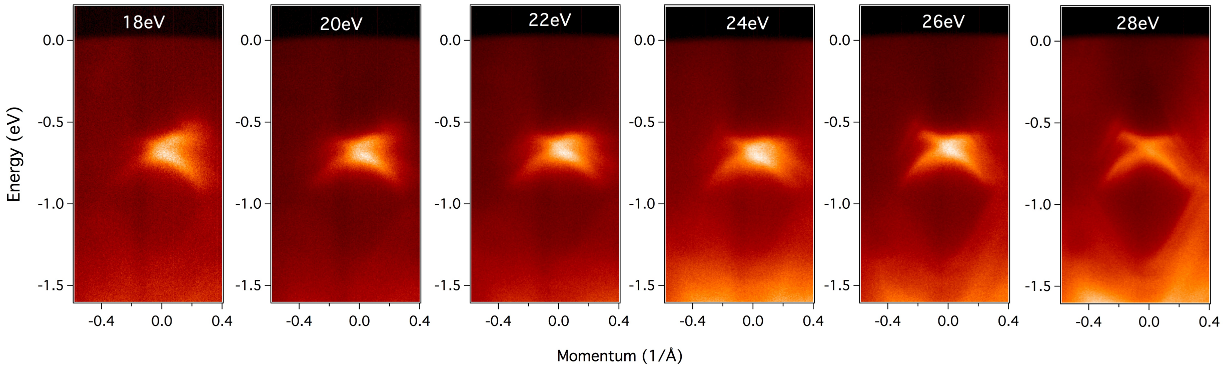

Supplementary Figure 2: Photon energy dependent spectra. ARPES dispersion maps measured at zone center Γ point with varying photon energy. The measured photon energy is noted on the spectra. These data were collected at SSRL beamline 5-4 at a temperature of 10 K.

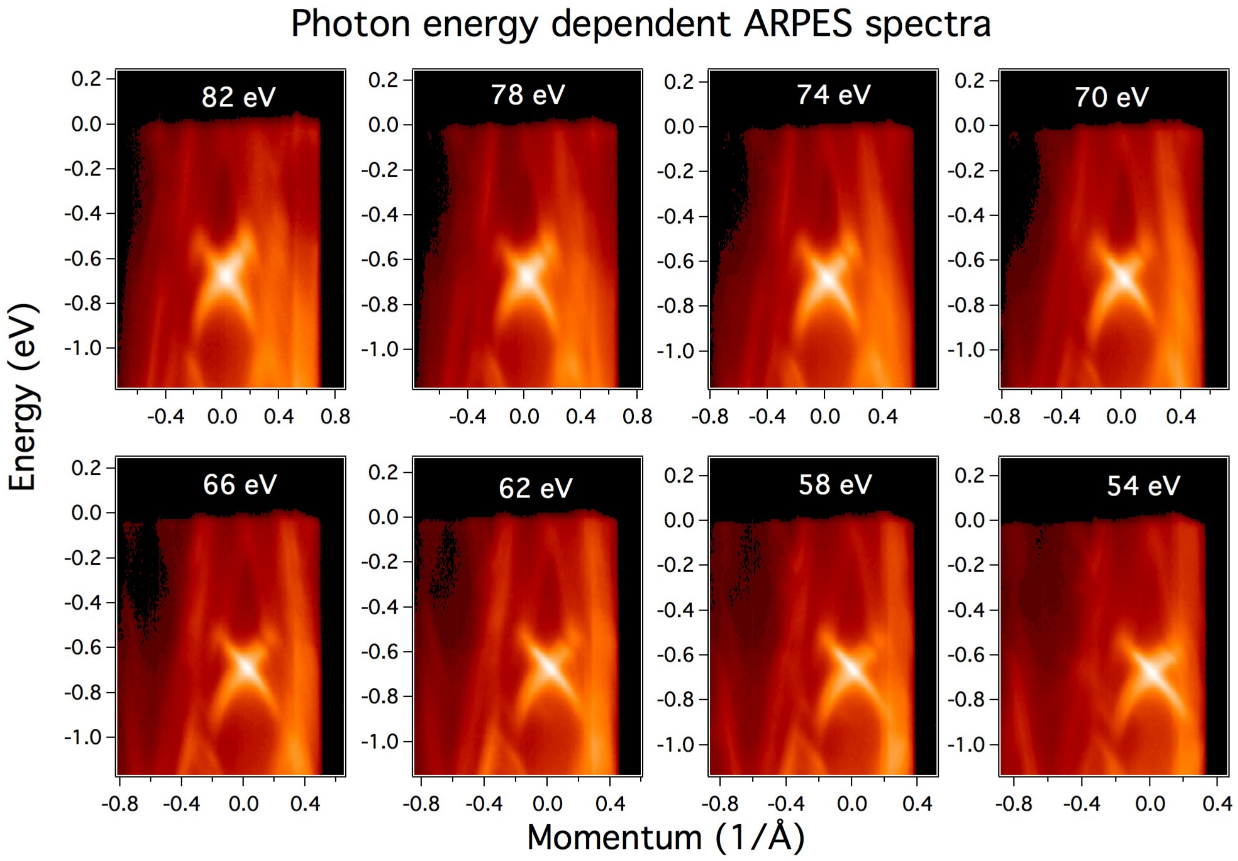

Supplementary Figure 3: Photon energy dependent ARPES measurements: Measured photon energies are marked on the plots.

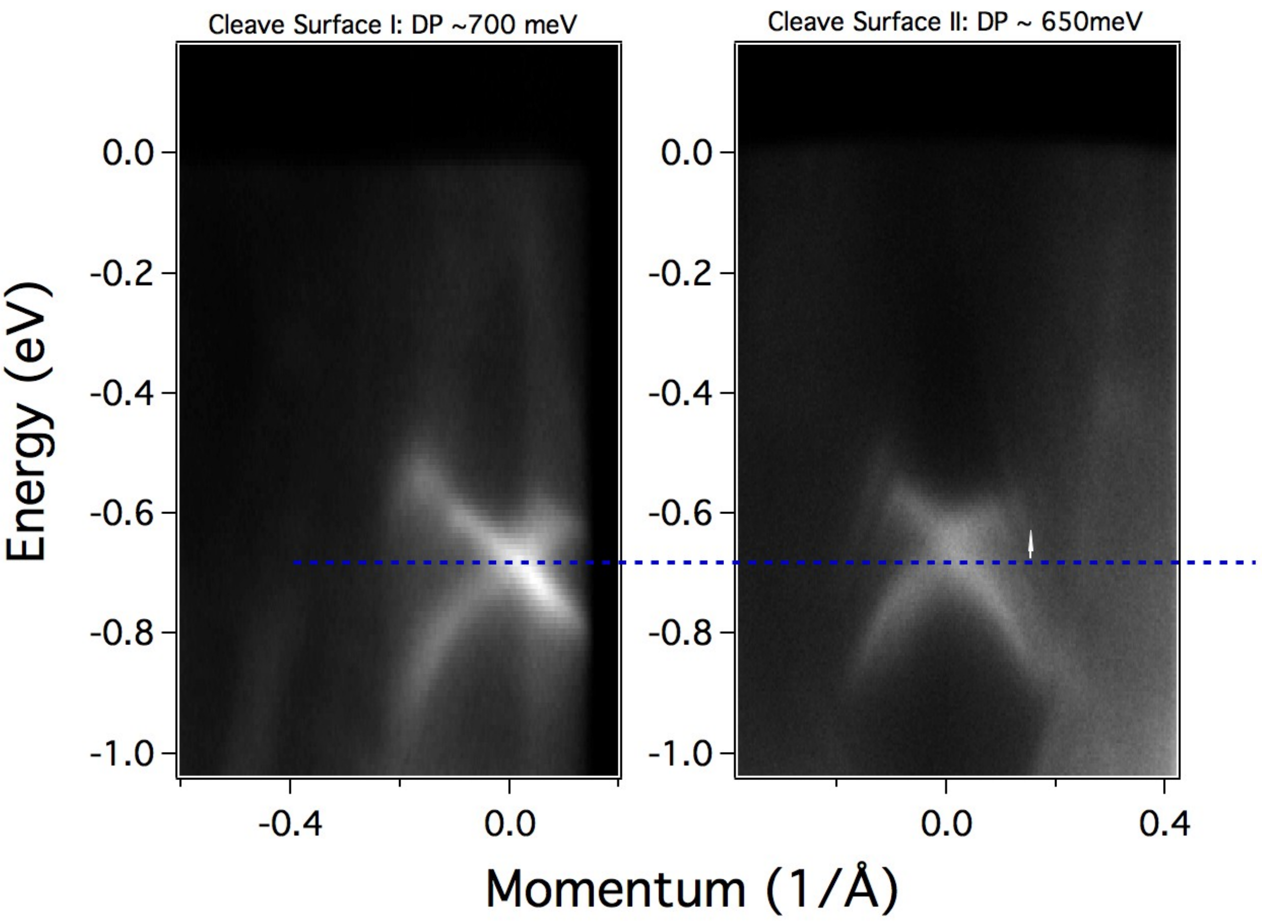

Supplementary Figure 4: ARPES energy momentum spectra obtained from two different cleaved surfaces. Blue dash line shows the energy position of the Dirac point for the cleaved surface I (left) and a small arrow shows the slightly different energy position for the cleaved surface II (right). These two spectra were measured in similar experimental conditions.

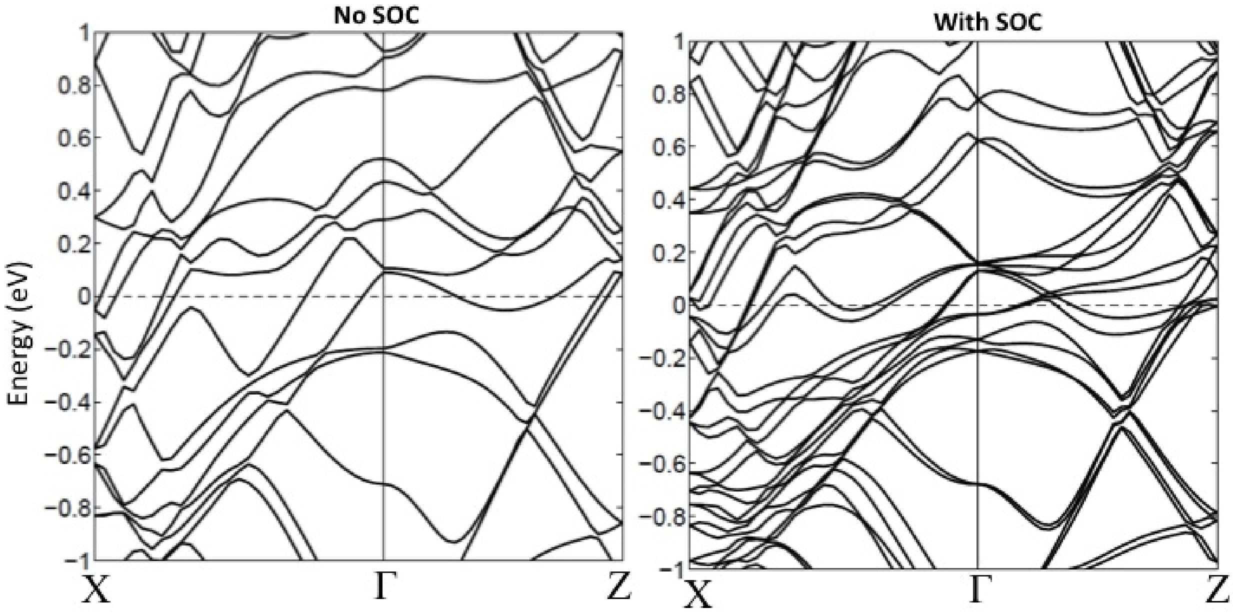

Supplementary Figure 5: Calculation results. Calculated bulk band structure with the projection along the (010) direction considering no spin-orbit coupling (left) and with spin-orbit coupling (right).

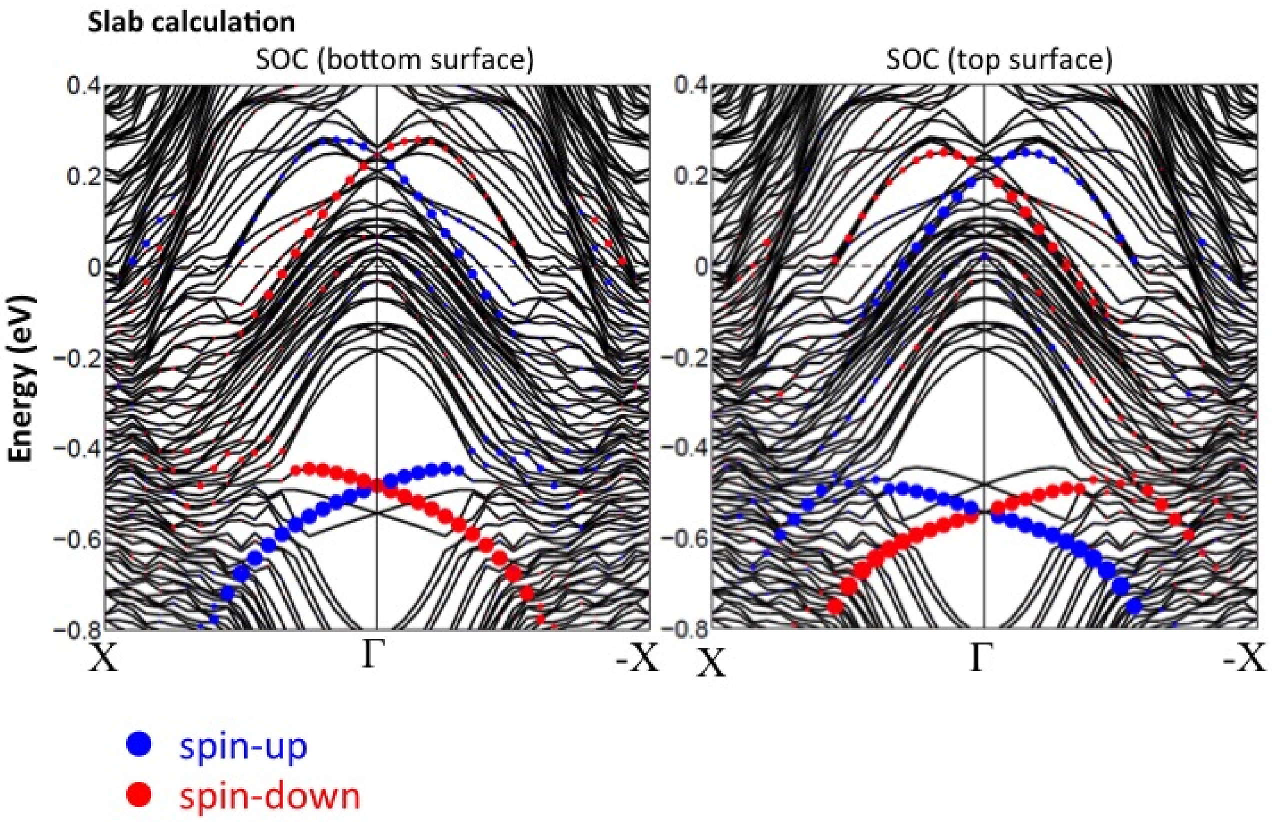

Supplementary Figure 6: In-plane spin polarization. Slab calculations at around the Γ point showing the helical in-plane spin structure of the surface band for the top surface (left) and bottom surface (right).

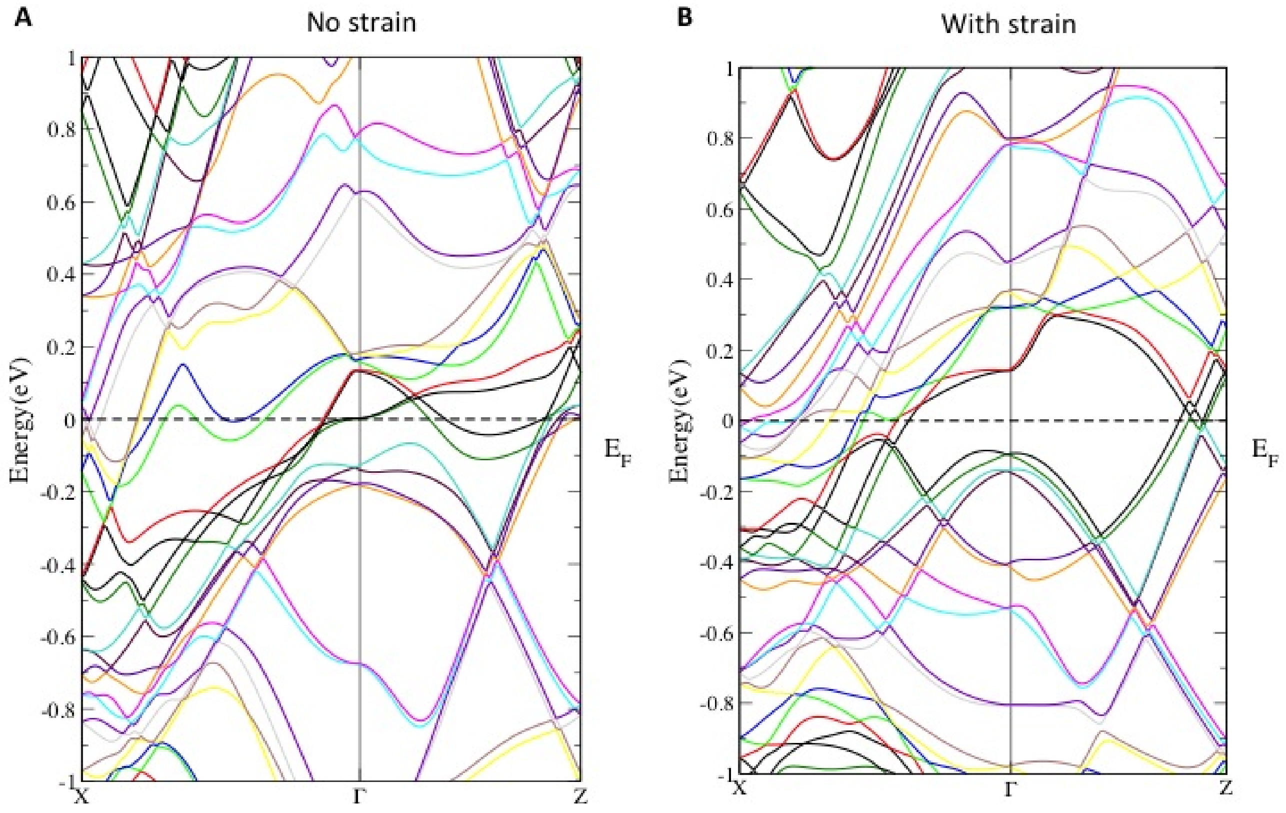

Supplementary Figure 7: (a) Band structure of BiPd without considering strain. (b) Band structure of BiPd with applying strain. We apply strain with b'=0.81b, a'=a/0.9, and c'=c/0.9 to keep the volume unchanged.

Supplementary Note 1

Sample characterization

Besides the Meissner state evidenced in the magnetic susceptibility data, the superconductivity in BiPd was proved by means of electrical transport and heat capacity measurements. The electrical resistivity was measured on a bar-shaped specimen with the dimensions 0.17 mm × 0.52 mm × 1.48 mm (the estimated error in geometrical factor is less than 10%) and the electrical contacts were made of silver epoxy paste. The measurement was performed with current flowing within the (010) plane. Fig. S1a displays the low-temperature electrical resistivity with a sharp drop to zero resistance at $T_c \sim 3.7$ K. It is worth noting that just above T_c, the resistivity is only $\rho_0 = 0.36$ $\mu\Omega$cm, and the ratio between the resistivity at room temperature and ρ_0 (residual resistivity ratio RRR) is as large as 113, which signals the high-quality of the single crystal studied. At the same temperature, the specific heat forms a distinct anomaly (see Fig. S1b), which corroborates the bulk nature of the superconducting state. The obtained results are in very good agreement with the literature data [1].

Supplementary Note 2

Photon energy dependent ARPES measurements

We present ARPES measured dispersion maps at the Γ point, with photon energies from 18 eV to 28 eV with every 2 eV energy step in Fig. S2. At a low photon energy, the bulk bands are suppressed and Dirac like surface states are highly enhanced. Most importantly, our wide range of photon energy measurements (from 18 eV to 82 eV) reveal a negligible dispersion of the Dirac like states confirming its two-dimensional nature as shown in Ref. [2, 3].

To further confirm the nature of the states in BiPd, we cleaved another sample. The ARPES dispersion map of this sample is shown in Fig. S3. ARPES spectra were measured from 56 eV to 82 eV with every 4 eV energy step. These data show that there is no clear dispersion with photon energy, which suggests its two-dimensional nature.

Since the calculations were made for slab geometry, the obtained band structure always involves bands from two surfaces, one on the top and the other on the bottom of the slab.

Experimentally, one can only measure one surface (corresponding to either top or bottom surface of the slab geometry) at a time. In principle, it should be possible to measure either top or bottom surface on the two sample pieces based on the cleaving. Therefore, depending on whether the cleaved surface is a top or bottom one, the location of the Dirac point energy is predicted to be different.

To approach both the top and bottom surfaces, we cleaved multiple samples and measured in identical experimental conditions such as sample temperature, photon energy, and the light polarization etc. Experimentally, the location of the Dirac point energy is observed to be different based on the cleaving of either the top or bottom surface, which is consistent with our calculations. We observed a different Dirac point energy for the top and bottom surfaces, based on the cleaving. For the top surface, the Dirac point is located at about 700 meV from the Fermi level, whereas the Dirac point is located at about 650 meV from the Fermi level for the bottom surface (see Fig. S4). It is a direct consequence of the centrosymmetric behavior of BiPd. We note that we cleaved multiple samples and most of cleaved surfaces show the Dirac point located at about 700 meV. We note that such two terminations of the crystal surface are directly observed by STM topographic image [4].

Supplementary Note 3

Calculations

Figures S5 and S6 show the calculated band structure along the high symmetry lines. In Fig. S5, the bulk band structure is shown along various high-symmetry lines without considering spin-orbit coupling (left) and with considering spin-orbit coupling (SOC) (right). These results are in agreement with Ref. [4]. SOC leads to the spin splitting of the bands as well as the shifting the energy position of the bands. The orbital character of the bands near the Fermi level is mostly contributed from Bi 6*p* and Pd 4*d* and they hybridized strongly leading to the complex Fermi surface as shown in Fig. 2 of the maintext.

Furthermore, our slab calculations show a helical in-plane spin structure (see Fig. S6) of the surface bands at the Γ point, which suggests that the surface states we experimentally observed in our ARPES measurements may be topological in nature. Spin calculation is consistent with the results obtained with spin-resolved ARPES measurements.

Our systematic experimental data and calculations reveal the following important prop-

erties of the electronic structure in BiPd:
(1) The bands located around the binding energy range of 900 meV to 500 meV are found to exhibit nearly linear (Dirac like) in-plane dispersion.
(2) No observable out-of-plane (k_z) dispersion is observed for these Dirac like bands.
(3) Our spin-resolved ARPES results and slab calculations show the helical spin structure of these surface states.
(4) We found an odd number of spin-momentum locked constant energy contour per Brillouin zone. This implies that if the Fermi level is tuned in the vicinity of the Dirac point, the odd number of Fermi surfaces formed by spin-momentum locked states can be found.
(5) The spin-momentum locked states wind around the time-reversal invariant momentum point.

All these above properties suggest the possibility of the topological surface state in BiPd. However, we caution that such properties are also present in noncentrosymmetric materials with high values of spin orbit coupling.

Supplementary Note 4

Effect of strain

We note that in ARPES experiments, the samples were cleaved and measured in the ultrahigh vacuum environment that kept the surface clean during the measurements. The sample cleaving process generates surface potential due to the surface charges, which may develop a large effective pressure along the *b* direction (perpendicular to the surface) and thus drive the crystal (possibly the several top layers) in the gapped state. It may be one of the possible reasons for the topological insulating behavior observed in BiPd. Such an argument is also suggested by a recent work in another noncentrosymmetric system [5]. We have done calculations with varying lattice constant *b*, keeping total volume constant. New calculations show that strain can open up the bulk band gap.

Here we used a full-potential linearized augmented plane wave method as implemented in the WIEN2k code [6]. The generalized gradient approximation [7] was also used for the exchange-correlation functional. The spin-orbit coupling was included in a second variational way. The muffin-tin radius $2.08a_0$ (a_0 being the Bohr radius), and $2.35a_0$ and for Bi and Pd, respectively, and a plane wave cutoff $RK_{max} = 8$ were taken in calculations that included

$11 \times 7 \times 11$ k-points. We note that the result for BiPd without strain from full-potential electronic structure calculations (left panel in Fig. S7) and that from the pseudopotential electronic structure calculations (right panel in Fig. S5) are quite consistent. In the case of compressive strain along the b direction, there is a clear tendency to open up a gap (see right panel in Fig. S7). In other word, the gap region is increased due to the strain applied on BiPd system.

Supplementary References

[1] Joshi, B. *et al.* Superconductivity in noncentrosymmetric BiPd. *Phys. Rev. B* **84**, 064518 (2011).

[2] Xia, Y. *et al.* Observation of a large-gap topological-insulator class with a single Dirac cone on the surface. *Nat. Phys.* **5**, 398 (2009).

[3] Neupane, M. *et al.* Surface electronic structure of the topological Kondo-insulator candidate correlated electron system SmB_6. *Nat. Commun.* **4**, 2991 (2013).

[4] Sun, Z. *et al.* Dirac Surface States and Nature of Superconductivity in Noncentrosymmetric BiPd. *Nat. Commun.* **6**, 6633 (2015).

[5] Bahramy, M. S., Yang, B.-J., Arita, R., and Nagaosa, N. Emergence of non-centrosymmetric topological insulating phase in BiTeI under pressure. *Nature Commun.* **3**, 679 (2012).

[6] Blaha, P. *et al.* An Augmented Plane Wave + Local Orbitals Program for Calculating Crystal Properties (K. Schwarz, Tech. Universitat Wien, Austria, 2001).

[7] Perdew, P., Burke, K., and Ernzerhof, M. Generalized Gradient Approximation Made Simple. *Phys. Rev. Lett.* **77**, 3865 (1996).

Correspondence and requests for materials should be addressed to M.N. (Email: Madhab.Neupane@ucf.edu).
